# Infective Endocarditis Due to *Corynebacterium jeikeium*: Four Case Reports and Narrative Review of the Literature

**DOI:** 10.3390/microorganisms12071337

**Published:** 2024-06-29

**Authors:** Daniel Arnés-García, Laura Lucena-Torres, Antonio Bustos-Merlo, Antonio Rosales-Castillo, Carmen Hidalgo-Tenorio

**Affiliations:** 1 Servicio de Medicina Interna, Hospital Universitario Virgen de las Nieves, 18014 Granada, Spain; lucena14795@gmail.com (L.L.-T.); antoniobustosmerlo@gmail.com (A.B.-M.); anrocas90@hotmail.com (A.R.-C.); 2 Unidad de Enfermedades Infecciosas, Hospital Universitario Virgen de las Nieves, Instituto de Investigación Biosanitario de Granada (IBS-Granada), 18014 Granada, Spain

**Keywords:** *Corynebacterium jeikeium*, infective endocarditis, antibiotic resistance

## Abstract

*Corynebacterium jeikeium*, a pleomorphic Gram-positive bacillus, is a common component of the cutaneous microbiota, usually considered as a contaminant, with little pathogenic potential. However, its role in various types of infections, such as bacteremia, sepsis, endocarditis (IE) and infection of prosthetic material is gradually being proven. Few cases of IE due to *Corynebacterium jeikeium* have been described in the literature. The aim of this article was to describe four cases of IE due to *Corynebacterium jeikeium* diagnosed in our hospital between May 2021 and April 2022, as well as to conduct a narrative review of the literature on this entity. After analysis, we highlight that 65.6% were men, 81.3% were valve or intravascular device carriers, and IE cases presented early, before one year after surgery. The most affected valve was the aortic valve (68.8%), followed by the mitral valve (21.1%). Valve replacement was performed in 65.6% of cases, and the most commonly used antibiotic was vancomycin (68.8%) at a dose of 15 mg/kg/12 h. With respect to prognosis, the overall mortality rate was 21.9%. The comparative results between our series and the literature review were similar except for a higher mortality rate (50%) and the use of dalbavancin in the treatment. We go on to review previously reported cases, along with four cases described in our hospital, of *C. jeikeium* endocarditis and will discuss various aspects of *C. jeikeium* infection, focusing on microbiology, pathophysiology, and treatment.

## 1. Introduction

*Corynebacterium jeikeium* is a coryneiform, catalase-positive and strictly aerobic Gram-positive bacillus [1]. Upon microscopic examination, they are observed forming clusters arranged in a palisade pattern, resembling Chinese characters. *C. jeikeium* belongs to the group of lipophilic corynebacteria, meaning they grow better in the presence of certain lipids, such as Tween 80. More than 80 species of *Corynebacterium* have been identified, and of these, more than 50 species have been linked to human diseases. The differentiation between Corynebacteriae species is carried out through certain studies such as Matrix-assisted laser desorption/ionization time-of-flight mass spectrometry (MALDI-TOF) or 16S rRNA gene sequencing. This species is part of the normal microbiota of the skin [2], being particularly abundant in the axillary, rectal, and inguinal regions of hospitalized patients. Although its isolation in blood cultures has been commonly considered as a contaminant [3], there are an increasing number of reports of true bacteremia and endocarditis (IE) [4,5]. In fact, it has been associated with different types of infection related to hospitalisation, broad-spectrum antibiotic treatment, breaks in the skin barrier, or the presence of a vascular catheter, mainly in patients with some type of immunosuppression, such as human immunodeficiency virus infection or neutropenia. Apart from the aforementioned vascular infections, pulmonary involvement, meningitis and infection of joint prostheses and cerebrospinal fluid shunts have also been reported. IE secondary to *C. jeikeium* most commonly affects left-sided heart valves and has a higher likelihood to require valve replacement. Moreover, it is associated with a high related mortality rate, which exceeds 30% despite correct treatment [6]. *C. jeikeium* presents multi-resistance to penicillins, cephalosporins and aminoglycosides; sensitivity to glycopeptides; and variable susceptibility to tetracyclines, rifampicin and quinolones [1]. An alternative regimen with high clinical effectiveness due to its dosing schedule and prolonged elimination half-life is the use of dalbavancin in the treatment of endocarditis and osteoarticular infections caused by *C. jeikeium*.

Here we present a series of cases of IE due to *C. jeikeium* from a tertiary hospital and compare it with cases published in a narrative review of the literature.

## 2. Materials and Methods

**Figure 1:**
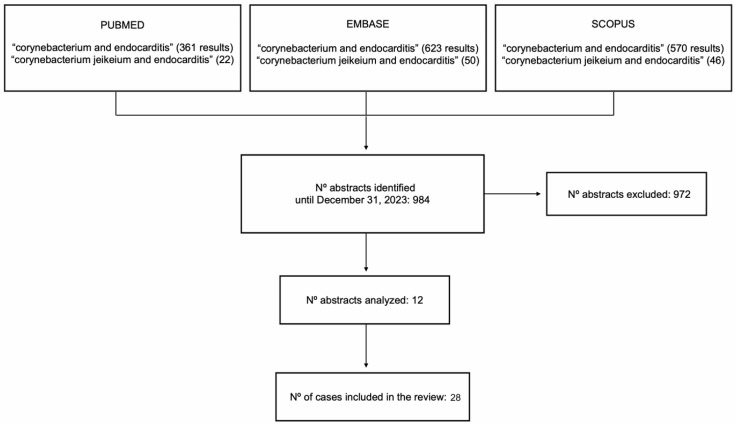
Flow chart of study selection in narrative review.

A search was made of articles published in English and Spanish up to 31 December 2023, in the PubMed-MEDLINE, Embase, and Scopus databases. Cases without a diagnosis of infective endocarditis or produced by corynebacteria other than *C. jeikeium* were excluded, as well as those articles whose content was not accessible or were written in a language other than English or Spanish. The search terms were “Corynebacterium and endocarditis” and “*Corynebacterium jeikeium* and endocarditis”. The final review included 12 articles containing 28 cases of *C. jeikeium* endocarditis. Figure 1 details the selection and inclusion process of the different studies.

Due to the descriptive, observational, retrospective and non-interventional nature of the study, a waiver of informed consent was accepted. Data were anonymized prior to analysis, in accordance with the Declaration of Helsinki and Spanish data protection legislation (Law 3/5 December 2018).

A descriptive statistical analysis was performed, calculating absolute and relative frequencies for qualitative variables, measures of central tendency and dispersion for quantitative variables, mean and standard deviation for those following a normal distribution, and median with IQR for those not following normality. Qualitative variables were compared using the chi-square test. A *p*-value < 0.05 was considered significant. The data were analysed using IBM SPSS Statistics 19.0 software (Armonk, NY, USA).

### 2.1. Case Series

In the following, we present the four cases of IE due to *C. jeikeium* diagnosed and treated in our centre between May 2021 and April 2022.

Case 1: A 70-year-old man was admitted for fever of unknown origin. Twenty-eight days ago, he underwent aortic valve replacement due to moderate stenosis (Perceval bioprosthesis). The patient had a history of essential arterial hypertension, type 2 diabetes mellitus, chronic obstructive pulmonary disease, atrial fibrillation and a single-chamber pacemaker due to atrioventricular block. After a transthoracic echocardiogram (TTE) without abnormal findings, a transoesophageal echocardiogram (TEE) was performed, showing native tricuspid and prosthetic aortic large vegetations, with reduced opening movement and thickening of the pacemaker wire. The pacemaker lead was removed and a double valve replacement was performed (aortic and tricuspid). *C. jeikeium* was isolated in peripheral venous blood cultures as well as in the pacemaker lead and valve cultures, with the following antibiogram (MIC in µg/mL): resistant to penicillin, moxifloxacin and clindamycin and sensitive to gentamicin (0.047), vancomycin (0.38), tetracycline (0.094), linezolid (0.25) and rifampicin (0.003). He received treatment with daptomycin at 12 mg/kg/24 h, rifampicin at 600 mg/24 h for six weeks, and gentamicin at 3 mg/kg/24 h for the first two weeks. The patient died four months later due to complications derived from valve replacement surgery: sternotomy dehiscence, mediastinitis due to *Candida albicans* and de novo severe mitral regurgitation, without echocardiographic or microbiological confirmation of infective endocarditis.

Case 2: A 64-year-old male patient was admitted for an intermittent fever evolving over two weeks, thirty-eight days after undergoing aortic valve replacement due to severe stenosis (Edwards Pericardial aortic bioprosthesis). He had a history of essential arterial hypertension, type 2 diabetes mellitus, hypercholesterolemia, obstructive sleep apnoea, and obstructive hypertrophic myocardiopathy. A TTE was performed, with a possible filamentous vegetation in the aortic valve that was confirmed by TEE. *C. jeikeium* was identified in two peripheral venous blood cultures with the following antibiogram (MIC in µg/mL): resistant to penicillin, moxifloxacin, fosfomycin and clindamycin, and sensitive to vancomycin (0.5), dalbavancin (0.047), daptomycin (3), linezolid (0.38) and rifampicin (0.004). He received antibiotic treatment for four weeks with linezolid 600 mg/12 h and daptomycin 12 mg/kg/24 h, continuing outpatient parenteral antibiotic treatment for two weeks more with dalbavancin at 1500 mg (two doses). One month after finishing antibiotic treatment, peripheral venous blood cultures were negative, and a control transthoracic echocardiogram was performed without findings. Unfortunately, one month later, he was admitted for ischemic stroke due to complete occlusion of the right vertebral artery and died as a result of its complications. No microbiological or echocardiographic recurrence of infective prosthetic endocarditis was observed.

Case 3: A 61-year-old male patient was admitted for fever and oligoarthritis thirty-seven days after undergoing aortic valve replacement surgery for severe stenosis (ATS mechanical prosthesis). He had a history of essential arterial hypertension and obstructive sleep apnoea. After an unremarkable TTE, a TEE was performed and showed a thickening of the posterior aortic wall suggestive of hematoma, although infective endocarditis could not be ruled out. *C. jeikeium* was isolated in serial peripheral venous blood cultures with the following antibiogram (MIC in µg/mL): resistant to penicillin, moxifloxacin and clindamycin and sensitive to vancomycin (0.38), daptomycin (0.38) and gentamicin (0.094). The patient received daptomycin 12mg/kg/24 h and rifampicin 600 mg/24 h for 14 days, continuing outpatient parenteral antibiotic treatment for four weeks with dalbavancin at 1500 mg (three doses). One year later, he remained asymptomatic with no echocardiographic or microbiological data of endocarditis recurrence.

Case 4: A 74-year-old female patient was admitted for fever of unknown origin forty-five days after undergoing aortic valve replacement due to severe regurgitation (Sorin Bicarbon mechanical prosthesis). She had a history of essential arterial hypertension and hypercholesterolemia. As she had a recent normal TTE, a TEE was directly performed, showing vegetation in the aortic prosthetic valve and pseudoaneurysm of the mitral-aortic intervalvular fibrosa. *Corynebacterium jeikeium* was identified in serial peripheral venous blood cultures with the following antibiogram (MIC in µg/mL): resistant to penicillin, moxifloxacin, fosfomycin and clindamycin and sensitive to gentamicin (0.047), vancomycin (0.38), dalbavancin (0.047), daptomycin (0.75), linezolid (0.75), daptomycin (0.75), linezolid (1.5) and rifampicin (0.003). She received antibiotic treatment with daptomycin at 10 mg/kg/24 h and linezolid at 600 mg/12 h. The patient underwent aortic valve replacement and reparation of the mitroaortic junction with a bovine pericardial patch. Unfortunately, a few days later, the patient died due to the development of a vasoplegic syndrome (distributive shock refractory to vasoactive drugs).

### 2.2. Literature Review and Discussion

*Corynebacterium jeikeium* is a saprophytic microorganism of the skin, colonizing areas such as the groin and armpits [7]. Although traditionally its microbiological isolation has been considered contamination [3], there is increasing evidence of its role as pathogen, producing anything from bacteremia and endocarditis to osteoarticular [8] and pulmonary infections [9]. Focusing on, I.E.; as it is well known, it is a serious entity secondary to the infection of the endocardial surface of the heart, usually referring to infection of one or more heart valves or infection of an intracardiac device. Risk factors include history of prior, I.E.; pre-existing valvular or congenital heart disease, intravenous drug use, cardiac device, intravenous catheter, immunosuppression or a recent dental or surgical procedure. Its clinical manifestations are highly variable, as is its form of presentation, ranging from acute and fulminant to chronic paucisymptomatic forms. The most common form of presentation is a febrile illness (up to 90%), often associated with shivering and constitutional symptoms such as hyporexia, malaise, weight loss or night sweats. Less frequent are immunological manifestations (rheumatoid factor positivity, Osler nodules, retinal Roth spots) and vascular manifestations (septic embolisms, intracranial or conjunctival haemorrhage, Janeway lesions…). With regard to causative microorganisms, it is important to distinguish between endocarditis on a native valve or a prosthetic or cardiac device as there are important differences at the microbiological level. In the former, the most important are *Staphylococcus aureus, Staphylococcus lugdunensis*, *Enterococcus faecalis*, streptococcal species and HACEK group microorganisms (*Haemophilus* species, *Aggregatibacter actinomycetemcomitans*, *Cardiobacterium hominis*, *Eikenella corrodens*, and *Kingella kingae*). However, in the case of the presence of a prosthesis or cardiac device, the following should also be included: coagulase negative staphylococci, *Corynebacterium* spp., *Cutibacterium acnes* and Gram-negative bacilli such as *Serratia marcescens* or *Pseudomonas aeruginosa*.

Few cases of endocarditis caused by *C. jeikeium* have been described in the literature. Table 1 shows the cases described in the medical literature so far (*n* = 28) that met the selection criteria detailed above. We have added to the table our series of cases presented in the previous section (*n* = 4). In total, 32 cases of IE due to C. jeikeium were collected.

The patients had a mean age of 58.8 years with a standard deviation of 13.7 years; 65.62% of the patients were male, and the acquisition of the infection was mainly nosocomial, having confirmed the colonization and intrahospital transmission of this pathogen [10]. Risk factors for IE due to *C. jeikeium* include immunosuppression and, given its ability to form biofilms [11], the presence of intravascular devices and prosthetic valves [3]. Among the selected cases of IE due to *C. jeikeium*, 28.13% (9/32) had intravascular devices and, in more than half of the cases (56.25%, 18/32), there was a history of cardiac valve replacement. As in our series, in patients with valve replacement surgery in whom the time since surgery was reported, infection occurred in all cases within the first year.

The diagnosis of IE is given by the modified diagnostic criteria of the European Society of Cardiology of 2023 [12]. Although TTE is a first approximation, its sensitivity is much lower than TEE, as shown by the data from our series, where TTE showed no alterations in two of the three cases of endocarditis. This diagnostic difference is especially marked in the case of prosthetic endocarditis, where TEE provides a better characterization of infectious lesions and local complications, detecting the presence of vegetations in more than 90% of cases. TEE is also indicated if transthoracic examination has revealed signs of valvular insufficiency, ventricular dysfunction, or extension of the infection to the valvular ring. On the other hand, new imaging techniques such as SPETC (single photon emission computed tomography) and PET/CT (positron emission tomography/computed tomography) or cardiac computed tomography (CT) also form part of the diagnostic criteria in the latest clinical guidelines [12]. Regarding microbiological diagnosis, the most widespread test of choice is the traditional blood culture, preferably serial. The isolation of *C. jeikeium* in a blood culture is more indicative of infection compared to the detection of other species of *Corynebacterium*, like *C. afermentans*, which are often contaminants [3]. Although their use is still limited, molecular biology techniques such as mass spectrometry (MALDI-TOF) [13] or PCR amplification of 16S ribosomal RNA [10] could allow microbiological identification, especially in cases of suspected IE with negative blood cultures or previous antibiotic treatment [14]. In one of the selected cases, microbiological isolation of *C. jeikeium* was achieved, in addition to blood cultures, by amplification of 16S ribosomal RNA in the perivalvular abscess [15]. This technique allows detecting the presence of *C. jeikeium*, distinguishing it from the rest of Corynebacterium species through the analysis of the 16S ribosomal RNA sequence. However, unlike blood cultures, it does not provide information on the antibiotic resistance pattern of *C. jeikeium* isolate.

The described cases of endocarditis most frequently affect the left-sided heart valves and have a high likelihood of requiring valve replacement. The most frequently affected valve was the aorta (68.8%, 22/32) followed by the mitral valve (28.1%, 9/32). In four patients (12.5%), there was involvement of two valves (in three of them, the aorta and mitral; and in the remaining one, the aorta and tricuspid), and in two (6.3%), isolated pacemaker infection. Valve replacement surgery was performed in 65.6% (21/32, excluding pacemaker replacement) of the cases of *C. jeikeium* endocarditis. The overall mortality rate was 21.9% (7/32), with no significant differences between patients undergoing surgery and those treated conservatively: 18.2% (4/22) vs. 30% (3/10) (*p* = 0.256). Although in our series the mortality obtained was 50% (2/4), it should be interpreted with caution due to the small number of cases included. It should also be noted that the two patients in our series who underwent surgery, and finally died, had local infectious complications (multivalvular involvement in patient number 1, and mitroaortic pseudoaneurysm in patient number 4) that make the surgical technique more difficult and may explain a higher in-hospital mortality.

In terms of antibiotic sensitivity, *C. jeikeium* presents mechanisms of high resistance to penicillins, cephalosporins, lincosamides, macrolides and quinolones [16,17,18]. Glycopeptides (vancomycin, teicoplanin) are the most commonly used antibiotics due to their high in-vitro sensitivity [16,17,18], and can be used empirically in the treatment of bacteremia and endocarditis caused by *C. jeikeium*. Of the selected cases, 68.8% (22/32) were treated with vancomycin. Resistance to aminoglycosides, rifampicin and carbapenemics is variable [16,17,18,19]. Generally, sensitivity to linezolid [20], daptomycin [19,21] and tigecycline [22] is preserved. Daptomycin has a high in-vitro activity against *Corynebacterium jeikeium* [23], although its antibiotic efficacy is lower than against staphylococci. The presence of intrinsic factors might be contributing to the lower susceptibility of *C. jeikeium* to daptomycin [24]. Daptomycin is the antibiotic used in six of the patients (18.7%), including all of our case series; however, the clinical course of these patients should be closely monitored, since loss of sensitivity and even a high-level resistance of this microorganism after a short period of exposure to daptomycin has been described [25,26]. Finally, *C. jeikeium* has a high in-vitro susceptibility to dalbavancin (DBV) [27]. Furthermore, DBV has demonstrated in vitro potent activity against Gram-positive cocci biofilms such as *Staphylococcus* spp. [28] and *Enterococcus* spp. [29], which is useful in the treatment of endocarditis and osteoarticular infections. In fact, DBV is a reasonable alternative as a consolidation antibiotic treatment in patients with IE due to Gram-positive cocci, with high clinical effectiveness, which allows shortening hospitalization and reducing hospital costs [30]. In our series of patients, half of them completed antibiotic treatment with DBV, and both had a favourable clinical evolution, being the first two cases published in the medical literature of IE due to *C. jeikeium* treated with this long-acting antibiotic.

**Table 1:** Characteristics of cases of infective endocarditis due to *Corynebacterium jeikeium* reported in the medical literature.

Case	Ref.	Year	Gender	Age (yo)	Comorbidities	Indwelling Line	History of Valve Replacement	Duke Diagnostic Criteria	TTE/TEE	Site Infection	Antibiotic Resistance	Antibiotic Therapy	Antibiotic Duration	Surgical Treatment	Outcome
1	[31]	1988	Male	68	AoR	No	Ao surgery	NS	NS	Ao	NS	Vancomycin, Rifampicin	NS	No	Recovery
2	[32]	1989	Female	77	AoS, MiR	No	Ao-Mi surgery	NS	NS	Mi	NS	Vancomycin	6 weeks	Yes	Recovery
3	[32]	1989	Male	51	MiR	No	No	NS	NS	Mi	NS	Vancomycin, Gentamicin	6 weeks	Yes	Recovery
4	[32]	1989	Male	54	CKD (HD), MiR	HD catheter	No	NS	NS	Mi	NS	Vancomycin	10 weeks	No	Recovery
5	[32]	1989	Female	57	MiS, TrR, coronary bypass	No	Ao-Mi surgery and Tri anuloplasty	NS	NS	Mi	NS	Piperacilina, Nstilmicin, Erythromycin	NS	No	Death
6	[32]	1989	Male	45	AoS/AoR	No	Ao surgery	NS	NS	Ao	NS	Vancomycin	4 weeks	Yes	Recovery
7	[33]	1990	Female	32	CKD (HD)	HD catheter	No	NS	NS	Ao and Mi	NS	Vancomycin	4 weeks	No	Death
8	[34]	1991	Male	60	Hepatic cirrhosis (Denver shunt)	Permanent catheter	No	Fever, PBC, cutaneous emboli, image	Vegetation (TTE)	Tr	NS	Vancomycin	4 weeks	No	Recovery
9	[35]	1992	Female	56	Liver transplant, CKD (HD)	HD catheter	No	NS	NS	Ao	NS	Vancomycin	2 weeks	Yes	Recovery
10	[35]	1992	Female	56	Liver transplant	Central line and HD catheter	No	PBC, image	Vegetation and AoR	Ao	NS	Vancomycin, Ceftazidime	4 weeks	Yes	Recovery
11	[36]	1993	Male	41	Failed kidney transplant (HD)	No	No	NS	NS	Ao	NS	Vancomycin, Gentamicin	NS	Yes	Death
12	[37]	1994	Female	17	AoR	No	Ao surgery (10 yo) with reintervention (<1 year ago)	Fever, PBC, image	Vegetation (TEE)	Ao	NS	Vancomycin, Gentamicin, Rifampicin	NS	Yes	Recovery
13	[38]	2001	Female	63	Coronary bypass	Femoral cannulation	No	NS	NS	Ao	NS	Vancomycin, Gentamicin	4 days	Yes	Death
14	[39]	2002	Male	53	NS	HD catheter	Mi surgery	NS	NS	Mi	NS	Vancomycin, Rifampicin	6 weeks	No	Death
15	[40]	2005	Male	68	Acute myeloid leukaemia receiving chemotherapy	Central line	No	Fever, PBC, cutaneous emboli	Vegetation and AoR (TEE)	Ao	NS	Vancomycin, Rifampicin	4 weeks	No	Recovery
16	[6]	2006	Male	84	AoS	No	Ao surgery	NS	NS	Ao	NS	Vancomycin, Gentamicin	6 weeks	Yes	Recovery
17	[41]	2007	Male	66	DM-2, AH	No	No	NS	NS	Ao	NS	Vancomycin	7 weeks	No	Recovery
18	[42]	2011	Male	72	PCM, ANCA vasculitis	No	No	NS	NS	PCM	NS	Vancomycin, Doxycycline + Rifampicin	6 weeks	PCM replacement	Recovery
19	[43]	2012	Male	57	AoS	No	Ao surgery	NS	NS	Ao	NS	Daptomycin, Rifampicin, Ceftazidime	6 weeks	Yes	Recovery
20	[44]	2014	Male	49	CKD (HD)	No	No	NS	NS	Ao	NS	Vancomycin	6 weeks	Yes	Recovery
21	[45]	2019	Female	53	CKD (HD)	No	Ao surgery (3 months ago)	NS	Ao abscess, Mi vegetation and MiR	Ao and Mi	NS	Vancomycin	12 weeks	Yes	Recurrence (recovery after surgery)
22	NP [5]	2019	Female	NS	AF, MiR (rheumatic)	No	Mi surgery	NS	NS	Mi	NS	Daptomycin	6 weeks	Yes	Recovery
23	NP [5]	2019	Male	NS	Bicuspid Ao, AoR (rheumatic)	No	Ao surgery and Mi reparation	NS	NS	Ao and Mi	NS	Vancomycin, Ceftriaxone	6 weeks	Yes	Recovery
24	[46]	2019	Male	65	CKD (HD)	HD catheter	No	PBC, image, valve culture	Vegetation and AoR (TEE)	Ao	NS	Daptomycin, Rifampicin	6 weeks	Yes	Recovery
25	[3]	2019	Female	60	NS	NS	Ao surgery	PBC, image	Abscess (TEE)	NS	NS	NS	NS	Yes	Recovery
26	[3]	2019	Male	75	NS	NS	Ao surgery	PBC, image	Vegetation (TEE)	NS	NS	NS	NS	Yes	Recovery
27	[47]	2020	Male	50	AH, peripheral artery disease	No	No	Fever, valve culture, image	Vegetation and AoR (TEE)	Ao	Penicillin	Vancomycin	6 weeks	Yes	Recovery
28	[15]	2021	Male	66	AH, AoS, AVB (PCM), coronary heart disease	No	Ao surgery (2.5 months ago)	Fever, image, PBC, 16S r-ARN (perivalvular abscess) **	Abscess and vegetation (TEE)	Ao and Tr	Penicillin	Vancomycin, Linezolid	8 weeks	Yes	Recovery
29	*	2021	Male	70	AH, DM-2, COPD, AF, AVB (PCM), AoE	No	Ao surgery (1 month ago)	Fever, PBC, image, valve culture	Tri-Ao vegetations, thickening of PCM wire (TEE)	Ao, Tr and PCM	Penicillin, clindamycin, moxifloxacin	Daptomycin, rifampicin, gentamicina (2 weeks)	6 weeks	Yes	Death
30	*	2022	Male	64	AH, DM-2, OSAS, AoS	No	Ao surgery (1.5 months ago)	Fever, PBC, image	Ao vegetation (TTE)	Ao	Penicillin, clindamycin, moxifloxacin fosfomycin	Daptomycin, linezolid; switch to dalbavancin	8 weeks	No	Recovery
31	*	2022	Male	61	AH, OSAS, AoS	No	Ao surgery (1 month ago)	Fever, PBC, arthritis, image	Ao vegetation (TEE)	Ao	Penicillin, clindamycin, moxifloxacin	Daptomycin, rifampicin; switch to dalbavancin	8 weeks	No	Recovery
32	*	2022	Female	74	AH, AoR	No	Ao surgery (1.5 months ago)	Fever, PBC, image	Pseudo-aneurysm, Ao vegetation (TEE)	Ao	Penicillin, clindamycin, moxifloxacin fosfomycin	Daptomycin, linezolid	10 days	Yes	Death

List of abbreviations. NP: not published; NS: not specified; Ao: aortic; Mi: mitral; Tr: tricuspid; PCM: pacemaker; AoR: aortic regurgitation; AoS: aortic stenosis; MiR: mitral regurgitation; MiS: mitral stenosis; TrR: tricuspid regurgitation; CKD: chronic kidney disease; HD: haemodialysis; TTE: transthoracic echocardiogram; TEE: transoesophageal echocardiogram; PBC: positive blood cultures; DM-2: type 2 diabetes mellitus; AH: arterial hypertension; AF: atrial fibrillation; AVB: atrioventricular block; COPD: chronic obstructive pulmonary disease; OSAS: obstructive sleep apnoea syndrome. * Patients included in our cohort and presented above in the Section 2.1. ** Not part of modified diagnostic criteria of the European Society of Cardiology of 2023.

## 3. Conclusions

*C. jeikeium* is a minor but increasingly frequent etiology of infective endocarditis. Infective endocarditis due to *C. jeikeium* is usually nosocomially acquired and is associated with intravascular devices and prosthetic valves (especially in the first year after surgery). It often affects the left-sided heart valves, requiring valve replacement in up to two-thirds of published cases. The overall mortality rate of *C. jeikeium* endocarditis is greater than 20%, which is due to the clinical situation of the patient, the antibiotic resistance profile of this BGP, and the need for surgery to control IE. In this scenario DBV may represent an optimal and more convenient alternative for the consolidation treatment of infective endocarditis due to its pharmacokinetic characteristics and prolonged elimination half-life.

## Data Availability

The data presented in this study are available in the main text.
